# The Structural Deciphering of the α3 Helix Within ZmHsfA2’S DNA-Binding Domain for the Recognition of Heat Shock Elements in Maize

**DOI:** 10.3390/plants14131950

**Published:** 2025-06-25

**Authors:** Yantao Wang, Zhenyu Ma, Guoliang Li, Xiangzhao Meng, Shuonan Duan, Zihui Liu, Min Zhao, Xiulin Guo, Huaning Zhang

**Affiliations:** 1 School of Landscape and Ecological Engineering, Hebei University of Engineering, Handan 056000, China; m13080408199@163.com (Y.W.);; 2 Hebei Key Laboratory of Plant Genetic Engineering, Institute of Biotechnology and Food Science, Hebei Academy of Agriculture and Forestry Sciences, Shijiazhuang 050051, China; mazhenyuqqtt@163.com (Z.M.);

**Keywords:** maize, heat shock transcription factor, heat shock element, arginine

## Abstract

Heat shock transcription factor (Hsf) plays a pivotal role in regulating plant growth, development, and stress responses. Hsf activates or represses target gene transcription by binding to the heat shock element (HSE) of downstream genes. However, the specific interaction sites between Hsf and the HSE in the promoter remain unclear. In this study, the critical amino acid residues of ZmHsf17 and the paralogous ZmHsf05 involved in DNA binding were identified using molecular docking models, site-directed mutagenesis, and the electrophoretic mobility shift assay (EMSA). The results reveal that both ZmHsf17 and ZmHsf05 bind to the HSE of the *ZmPAH1* promoter via a conserved arginine residue located in the α3 helix of their DNA-binding domains. Sequence substitution experiments among distinct HSEs demonstrated that flanking sequences upstream and downstream of the HSE core synergistically contribute to the specificity of DNA-binding domain recognition. Comparative evolutionary analysis of DNA-binding domain sequences from 25 phylogenetically diverse species reveals that the α3 helix constitutes the most conserved structural element. This study elucidates the key interaction sites between maize HsfA2 and its target genes, providing theoretical insights into the binding specificity to the HSEs of the plant’s Hsf family and the functional divergence. Additionally, these findings offer new targets for the precise engineering of Hsf proteins and synthetic HSEs.

## 1. Introduction

Global warming-induced heat stress has emerged as a critical constraint on plant growth and agricultural productivity [1]. Elevated temperatures impair normal plant development through multifaceted mechanisms, including the disruption of proteostasis, the induction of membrane lipid peroxidation, and damage to photosynthetic systems [2]. To counteract thermal stress, plants have evolved sophisticated molecular networks wherein heat shock transcription factor (Hsf) plays a central regulatory role by orchestrating the expression of heat shock proteins (Hsps) and thermotolerance-related genes [3].

The transcriptional activity of Hsf is governed by coordinated interactions among conserved structural domains. A canonical transcriptionally active Hsf typically comprises three functional modules: an N-terminal DNA-binding domain (DBD), an adjacent oligomerization domain (OD), and a C-terminal activation domain characterized by aromatic, hydrophobic, and acidic amino acid residues (the AHA motif) [4]. Structural analyses reveal that the DBD, spanning 80–100 amino acids, adopts a winged helix–turn–helix topology comprising three α-helices (α1–α3) and four β-strands (β1–β4) [5]. Crucially, the third α-helix (α3), designated as the recognition helix, specifically interacts with the heat shock element (HSE) core motif “GAA” through insertion into the major groove of DNA, establishing sequence-specific binding competence [6].

Hsf is universally conserved across eukaryotic organisms, yet exhibits remarkable lineage-specific diversification in gene family size and functional complexity. Initial genomic surveys revealed stark contrasts: *Saccharomyces cerevisiae* and *Drosophila melanogaster* were each found to possess a single *Hsf* gene, while vertebrates expanded this repertoire to four paralogs (Hsf1, Hsf2, Hsf3 and Hsf4) through genome duplication events [7]. In striking contrast, terrestrial plants evolved large Hsf families, with the first members cloned from tomato (*Solanum lycopersicum*) demonstrating their essential role in thermotolerance [8]. Plant Hsfs are systematically classified into three evolutionary conserved classes (A, B, and C) based on structural and functional criteria. Among these, Class A members (A1, A2, A3, A6, A9) are particularly noteworthy for containing conserved transcriptional activation domains and showing rapid upregulation under heat stress, directly activating downstream targets such as HSP101 and APX2 [9]. Biochemical analyses in Arabidopsis established that HsfA1 subfamily members remain sequestered in the cytoplasm under non-stress conditions through interactions with cytosolic chaperones (Hsp70/90), forming inactive 450 kDa heterocomplexes [10]. Thermal perturbation triggers the competitive binding of misfolded proteins to Hsp70/90, liberating HsfA1 monomers that subsequently form nuclear-localized homo-oligomers via their ODs. This redox-sensitive regulatory switch enables rapid transcriptional reprogramming, with activated Hsf binding to conserved HSEs in target promoters [11]. Under heat stress, the DNA-binding activity of Hsfs to HSEs is markedly potentiated compared to its basal binding capacity observed at normal temperatures. The heat-induced phosphorylation of Hsf serves as the primary regulatory mechanism enhancing its DNA-binding competence [12]. Mechanistically, the Ca^2+^-CaM signaling axis activated by thermal stress orchestrates Hsf phosphorylation dynamics through coordinated kinase/phosphatase activation, thereby fine-tuning Hsf-DNA interaction kinetics [13].

Despite extensive investigations into the genetic functions and molecular mechanisms of Hsf, the regulatory roles of critical amino acid residues in modulating DNA-binding activity remain poorly characterized [14]. Notably, human Hsf4 governs sensory organ development (e.g., ocular lens formation), where natural mutations within its DBD are associated with congenital cataracts across ethnic populations [15]. Structural studies demonstrate that single amino acid substitutions in Hsf4-DBD abolish HSE recognition, highlighting the functional sensitivity of this domain. The evolutionary conservation of DBD architecture further underscores its functional specialization [16]. Hsfs predominantly function as homotrimers or heterohexamers, with multimerization enhancing DNA-binding affinity through synergistic interactions between adjacent DBDs [17]. This oligomerization process depends on the OD, an 80 amino acid domain featuring contiguous α-helical structures. In Arabidopsis, HsfA1d activates APX2 expression via trimer formation, and the substitution of cysteine residues critical for disulfide bonding in its OD disrupts transcriptional regulation, confirming the necessity of key oligomerization residues for functional assembly [18]. While these findings emphasize the importance of domain-specific residues, systematic identification of functional amino acids in plant Hsf remains limited.

In maize (*Zea mays*), the Hsf family comprises 31 members, with five A2 subfamily and one A6 subfamily genes exhibiting pronounced thermoresponsive upregulation [19]. Our preliminary study identified ZmHsf17, an A2 subfamily member, as a thermotolerance regulator that stabilizes membrane lipid homeostasis under heat stress by activating ZmPAH1 expression [20]. Intriguingly, interference with three DBD residues (Arg105, Thr109, and Lys142) by alternative splicing-derived peptides attenuates ZmHsf17’s DNA-binding capacity, suggesting their direct involvement in HSE recognition [21]. To mechanistically dissect these interactions, we first employed molecular docking to predict the binding interface between ZmHsf17 and the *ZmPAH1* promoter. Site-directed mutagenesis was subsequently applied to generate DBD variants, followed by prokaryotic protein expression and purification for functional validation. Electrophoretic mobility shift assays (EMSAs) were conducted to systematically map HSE-binding residues using both ZmHsf17 and its paralog ZmHsf05. Furthermore, sequence substitution analyses were performed to evaluate the contribution of HSE flanking regions to DNA recognition specificity. The phylogenetic reconstruction of the Hsf-DBD was conducted through the comparative sequence alignment of ZmHsf17 and ZmHsf05 against orthologous sequences from 24 phylogenetically representative taxa.

## 2. Results

### 2.1. Modeling Interaction Interfaces of ZmHsf17-DBD and ZmPAH1-HSE Using AlphaFold 3

**Figure 1:**
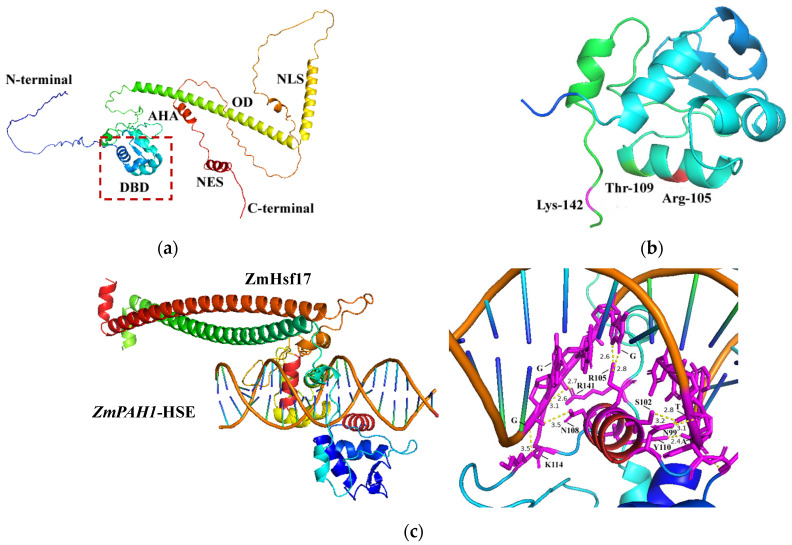
ZmHsf17 binds to the HSE motif in the promoter region of *ZmPAH1.* (**a**) A schematic diagram of the tertiary structure of ZmHsf17. Amino terminus: N-terminal, DNA-binding domain (red rectangle): DBD, oligo-binding domain: OD, nuclear localization signal: NLS, acidic amino acid residues: AHA, nuclear export signal: NES, carboxyl terminus: C-terminal. (**b**) The three protein interaction sites between ZmHsf17-DBD and the truncated short peptide. Red represents the arginine residue at position 105: Arg-105 (R105), green represents the threonine residue at position 109: Thr-109 (T109), and purple represents the lysine residue at position 142: Lys-142 (K142). (**c**) A schematic representation of the ZmHsf17 homodimer binding to the HSE of *ZmPAH1* promoter in Alphafold 3 (ipTM = 0.44; pTM = 0.74). ZmHsf17 monomers dimerize via their ODs, which adopt extended contiguous α-helical structures (the red and green cartoon representations). Within the DBDs, a short α helix (the red cartoon) inserts into the major groove of the target DNA. A magnified view of the protein–DNA interface reveals putative hydrogen bonds (the yellow dashed lines; bond length ≤ 3.5 Å) between specific amino acid residues and nucleotide bases, with the interacting moieties highlighted in magenta.

The tertiary structure prediction of ZmHsf17 based on its amino acid sequence revealed a canonical helix–turn–helix topology in the N-terminal DBD, an extended α-helix forming the OD, and short α-helical segments constituting the nuclear localization signal (NLS), AHA, and nuclear export signal (NES) domains (Figure 1a). Previous work from our laboratory demonstrated that truncated peptide ZmHsf17-II from alternative splicing inhibits the DNA-binding activity of ZmHsf17 itself, with Arg105 (R105), Thr109 (T109), and Lys142 (K142) identified as critical interaction sites [21]. Structural analysis localized R105 and T109 within the α3 helix, while K142 resides at the DBD terminal junction (Figure 1b). Modeling in AlphaFold 3 predicted specific interactions between ZmHsf17-DBD-OD homodimer and the double-stranded canonical HSE motif in the *ZmPAH1* promoter (Figure 1c). The ZmHsf17 dimer mediates sequence-specific recognition of the cis-acting HSE through the dual docking of its two DBDs to complementary strands of the double-stranded DNA motif. A magnified view of the binding interface revealed potential hydrogen bonding between the amino acid residues in the α3 helix and the HSE core sequence (Figure 1c). Structural modeling predicts that five residues (N99, S102, R105, N108, and Y110) within the α3 helix of the DBD potentially form hydrogen bonds with the DNA backbone (Figure 1c). R105 is one of the three key amino acids (R105, T109, and K142) previously reported to potentially affect ZmHsf17 binding activity. Notably, the lysine (K114) in the β3 strand and a C-terminal arginine (R141) of the DBD also engage in hydrogen bonding (Figure 1c).

### 2.2. R105A Substitution Impairs DBD-HSE Binding

**Figure 2:**
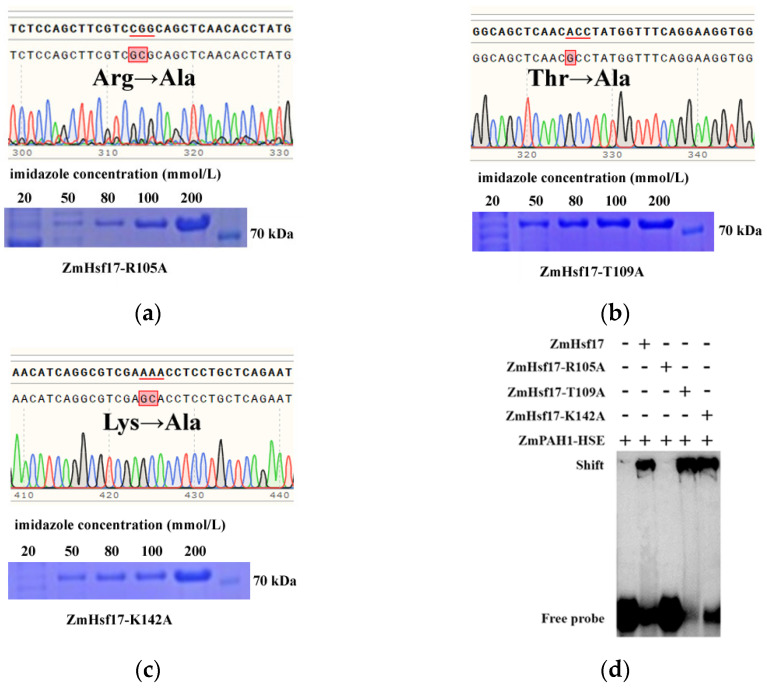
The amino acid site-directed mutagenesis of ZmHsf17 and the validation of binding to the HSE of *ZmPAH1* promoter. Sequencing peak charts of Arg105 (**a**), Thr109 (**b**), and Lys142 (**c**) mutated to alanine, and the SDS-PAGE electrophoresis results of elution fractions with different imidazole concentrations in in vitro purification (red underlines denote codons encoding the three target amino acid residues, while black arrows indicate site-directed alanine substitutions); (**d**) the EMSA of ZmHsf17 and its mutants binding to the HSE of the *ZmPAH1* promoter.

During the interaction modeling, we performed site-directed mutagenesis on the three amino acids (R105A, T109A, and K142A) of ZmHsf17. Three independent single-alanine substitution mutants were constructed via overlap extension PCR-mediated site-directed mutagenesis, with each mutation targeting individual residues in the DNA-binding domain (Figure 2a–c). Prokaryotic expression and purification yielded mutant proteins ZmHsf17-R105A, ZmHsf17-T109A, and ZmHsf17-K142A. Fusion proteins carrying His-tag were enriched the most in the elution solution with 200 mmol/L imidazole, and the molecular weight of the proteins is approximately 70 kDa (Figure 2a–c). The electrophoretic mobility shift assay (EMSA) demonstrated that ZmHsf17 bound the *ZmPAH1* HSE probes, forming distinct retarded complexes. Notably, the R105A mutation abolished HSE binding, whereas the T109A and K142A mutants retained DNA-binding capacity (Figure 2d). This residue-specific functional divergence underscores the pivotal role of R105 in controlling sequence-specific DNA recognition.

### 2.3. Conserved Arginine Function Validated in the Paralog ZmHsf05

**Figure 3:**
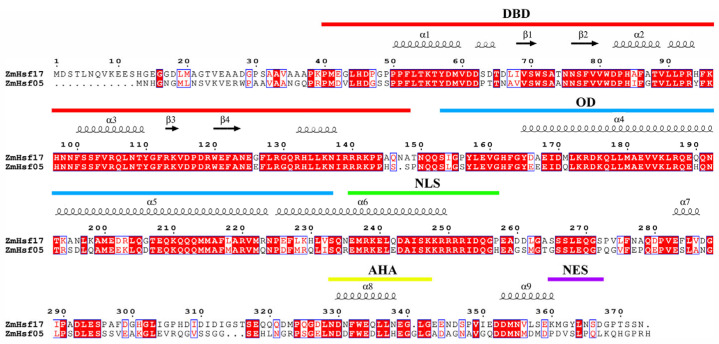
Alignment of the amino acid sequence of ZmHsf17 with ZmHsf05. The different color lines represent distinct functional domains within the protein architecture. The “α” and “β” denote α-helices and β-sheets, respectively, fundamental secondary structural elements of proteins.

**Figure 4:**
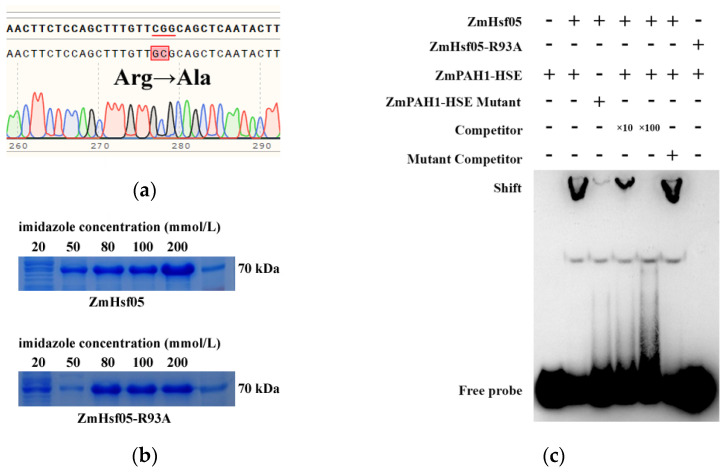
The amino acid site-directed mutagenesis of ZmHsf05 and the validation of binding to the HSE of the *ZmPAH1* promoter. (**a**) The sequencing peak charts of Arg93 mutated to alanine (red underlines denote codons encoding the three target amino acid residues, while black arrows indicate site-directed alanine substitutions); (**b**) the SDS-PAGE electrophoresis results of the ZmHsf05 and ZmHsf05-R93A elution fractions with different imidazole concentrations in in vitro purification; (**c**) the EMSA of ZmHsf05 and ZmHsf05-R93A binding to the HSE of the *ZmPAH1* promoter.

Previous studies identified ZmHsf05 and ZmHsf17 as paralogs originating from chromosomal duplication, with phylogenetic analyses confirming their high sequence similarity [22]. Sequence alignment revealed that both proteins retain conserved Hsf domains (DBD, OD, and AHA), with the highest conservation in the DBD, particularly within the α3 helix (Figure 3). Strikingly, the arginine residue corresponding to ZmHsf17-R105 is evolutionarily preserved as R93 in ZmHsf05, with identical flanking sequences in the α3 region. Overlap extension PCR-mediated site-directed mutagenesis generated ZmHsf05-R93A, and subsequent prokaryotic purification yielded ZmHsf05 and mutant proteins (Figure 4a,b). The EMSA demonstrated that ZmHsf05 bound the *ZmPAH1* HSE probes, while the R93A mutation completely abolished DNA binding (Figure 4c), confirming the indispensable role of this conserved arginine in paralogous HSE recognition.

### 2.4. Flanking Sequences Affect HSE Recognition Specificity

**Figure 5:**
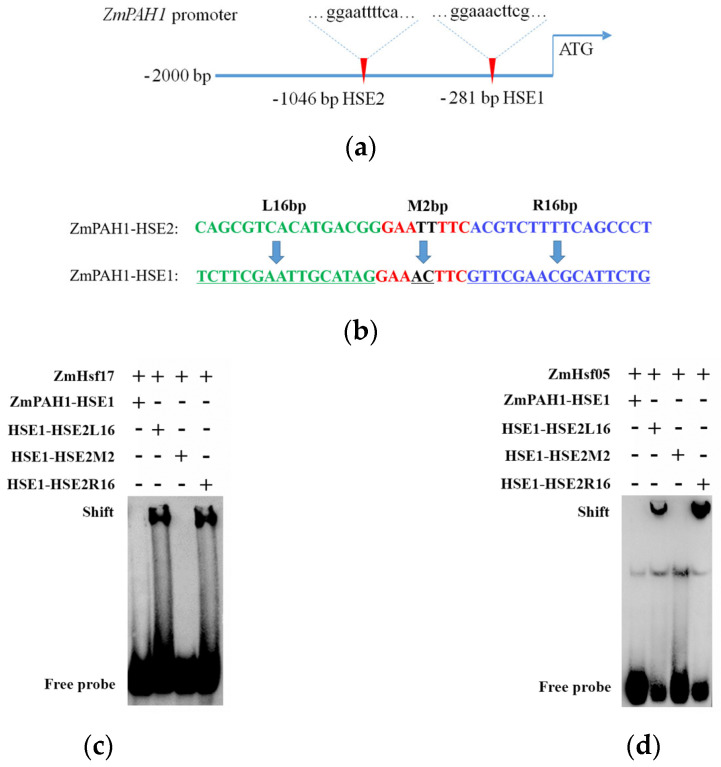
The flanking sequences upstream and downstream of different HSEs are essential for the specific binding of HsfA2 to the HSE. (**a**) The two HSEs located in the promoter region of *ZmPAH1*; (**b**) the upstream flanking sequence (L16bp), middle sequences between GAA repeats (M2bp), and downstream flanking sequence (R16bp) of HSE1 were, respectively, replaced by the sequences at the corresponding position of HSE2; (**c**,**d**) The EMSAs of ZmHsf17 (**c**) and ZmHsf05 (**d**) binding to ZmPAH1-HSE1 probes with partially replaced sequences.

Analysis of the *ZmPAH1* promoter (−1 to −2000 bp) identified three canonical HSE motifs (nGAAnnTTCn), designated as HSE1 and HSE2 based on their proximity to the transcription start site (Figure 5a). ChIP-seq data from prior work revealed ZmHsf17 exclusively bound HSE2, consistent with the EMSA results for both ZmHsf17 and ZmHsf05. Neither protein exhibited binding to HSE1 (Figure S1), suggesting sequence context beyond the core GAA repeats modulates Hsf-DNA specificity.

To dissect flanking sequence contributions, three chimeric HSE1 variants were engineered: HSE1-HSE2L16 (16 bp upstream flanking sequence from HSE2), HSE1-HSE2M2 (2 bp between GAA repeats from HSE2), and HSE1-HSE2R16 (16 bp downstream flanking sequence from HSE2) (Figure 5b). The EMSAs revealed that ZmHsf17 and ZmHsf05 bound HSE1-HSE2L16 and HSE1-HSE2R16 probes but failed to interact with HSE1-HSE2M2 (Figure 5c,d), indicating that flanking sequences at both termini but not the inter-GAA spacer critically determine recognition specificity.

### 2.5. Evolutionary Analysis of Hsf-DBD

**Figure 6:**
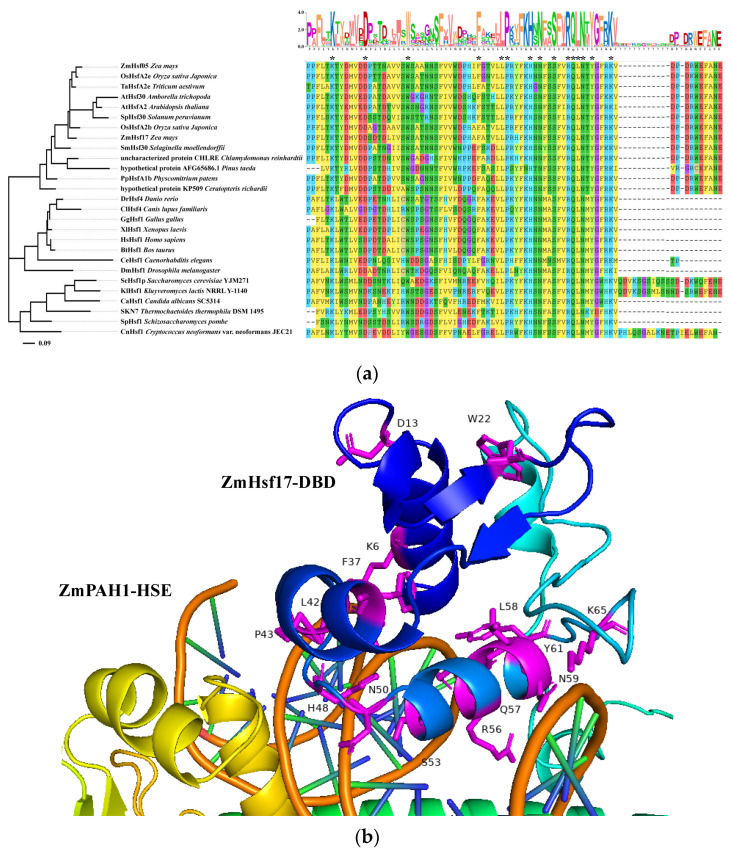
Amino acid sequence alignment and the phylogenetic tree of Hsf-DBD regions in 25 species. (**a**) Multiple sequence alignment of Hsf-DBDs across 25 species. Asterisks (*) denote residues identical in all species. Sequence logo of the conserved DBD motif (top panel), with the y-axis indicating conservation score in bits. Residue-specific conservation is quantified by letter height on the x-axis—larger single-letter amino acid codes reflect higher positional conservation. Positions marked with question marks (?) indicate insufficient sample size (<5 sequences) for statistical significance. The accession numbers of the Hsf-DBD-containing proteins from 24 additional species are listed below. *Oryza sativa Japonica* HsfA2e: Q6F388.1; *Oryza sativa Japonica* HsfA2b: Q6VBB2.1; Triticum aestivum: XP_044383382.1; *Arabidopsis thaliana* HsfA2: O80982.1; *Solanum peruvianum*: P41152.1; *Amborella trichopoda*: XP_006853865.1; *Arabidopsis thaliana* HsfA6b: Q9LUH8.1; *Physcomitrium patens*: XP_024382543.1; *Ceratopteris richardii*: KAH7331940.1; *Selaginella moellendorffii*: XP_002977898.2; *Homo sapiens*: KAI2551788.1; *Bos taurus*: Q08DJ8.1; *Gallus gallus*: P38529.1; *Xenopus laevis*: P41154.1; *Danio rerio*: Q5CZP2.2; *Canis lupus familiaris*: Q1HGE8.1; *Drosophila melanogaster*: P22813.1; *Caenorhabditis elegans*: G5EFT5.1; *Cryptococcus neoformans*: Q5KMX8.1; *Schizosaccharomyces pombe*: Q02953.2; *Thermochaetoides thermophila*: G0SB31.1; *Candida albicans SC5314*: Q5AQ33.2; *Saccharomyces cerevisiae*: AJR79024.1; *Kluyveromyces lactis* NRRL Y-1140: P22121.1; *Chlamydomonas reinhardtii*: XP_001694420.2; *Pinus taeda*: AFG65686.1. (**b**) Close-up view of the predicted ZmHsf17-DBD and *ZmPAH1* promoter–HSE interaction interface in AlphaFold 3. ZmHsf17-DBD is presented in cartoon form. All fifteen highly conserved amino acid side chains are highlighted in magenta.

The phylogenetic reconstruction of the Hsf-DBD was performed by aligning the ZmHsf17 and ZmHsf05 sequences against 24 evolutionarily pivotal species spanning plants, animals, fungi, and thermophilic archaea. The DBDs of orthologous genes from 11 plant species, 8 animals, 5 fungi, and 1 thermophilic archaeon exhibited high sequence similarity (Figure 6a). Strikingly, a conserved tetrapeptide motif “RQLN” was identified in the α3 helix across all analyzed species (Figure 6a), confirming this region as the evolutionary core for DNA binding. The arginine residue (R) within the “RQLN” tetrapeptide was identified as a critical determinant of HSE binding, with both ZmHsf17-R105A and its paralog ZmHsf05-R93A exhibiting a complete loss of DNA-binding capacity in the EMSAs. Additional conserved residues critical for structural stability were observed, including lysine (K) in the α1 helix, tryptophan (W) in the β1 sheet, and phenylalanine (F), leucine (L), and proline (P) in the α2 helix (Figure 6a).

The phylogenetic tree topology revealed clear divergence between major taxonomic groups. A well-supported plant-specific clade retained the β4 sheet with high sequence conservation (D/VP/R-D/GR/KW/CEFANE) in this region (Figure 6a). In contrast, the animal-specific clade formed a distinct cluster lacking β4 sheet structures. Notably, *Chlamydomonas reinhardtii* and *Pinus taeda* clustered closer to the animal and fungal clade, displaying intermediate β4 sheet features with 100% and 60% sequence identity to core land plants (Figure 6a).

Within the structural interface model, all fifteen highly conserved amino acid residues were mapped for their spatial relationship with DNA. Notably, thirteen residues localize to the protein–DNA interaction surface, exhibiting close proximity to the double-stranded DNA backbone (Figure 6b). While the D13 and W22 resides were distal from direct DNA contact, their strategic positioning at the junctional regions of secondary structural elements suggests potential structural roles in maintaining tertiary conformation essential for DNA-binding competency (Figure 6b).

## 3. Discussion

As a central regulator of the heat shock response, Hsf orchestrates thermotolerance by forming homo- or hetero-oligomers that specifically bind HSEs in target promoters, thereby activating HSP and stress-responsive genes [3]. While the thermoprotective roles of Hsfs are well-documented, the molecular determinants governing DNA binding specificity within their DBDs remain underexplored. The conserved helix–turn–helix motif in DBDs enables precise recognition of the HSE core sequence (nGAAn), a universal mechanism preserved across eukaryotes [23]. Structural homology between the Arabidopsis HsfA1a and human Hsf1 DBDs underscores this evolutionary conservation, with both proteins activating Hsp genes despite divergent evolutionary trajectories [24]. Members of the HsfA2 subclass exhibit robust thermoresponsive upregulation under heat stress, enabling the rapid assembly of Hsf homomultimers [3]. Multiple DBDs in Hsf homomeric complexes with strong co-operativity cooperatively engage clustered HSEs, enhancing binding avidity through multivalent interactions [25]. Moreover, structural diversity within the C-terminal AHA motifs synergistically amplifies transcriptional activation capacity by recruiting distinct co-activators [26].

A conserved arginine-containing α3 helix functions as the recognition helix in both yeast and fruit fly Hsfs, mediating sequence-specific binding to the central guanine (G) within the HSE core motif [27]. Similarly, Arg63 in HSF2 or Arg71 in HSF1 in humans are indispensable for HSE recognition, as mutations abolish DNA binding [5,28]. These conserved mechanisms highlight the universal importance of arginine residues in DBD functionality, a paradigm corroborated by our findings in maize ZmHsf17. Our study bridges this evolutionary gap by identifying Arg105 in ZmHsf17-DBD as a critical mediator of HSE binding. Notably, this residue aligns spatially and functionally with Arg71 in human Hsf1 [28], suggesting the deep conservation of arginine-dependent DNA recognition across kingdoms. The loss of DNA-binding capacity in ZmHsf17-R105A mirrors the functional disruption observed in human Hsf1 arginine mutants, reinforcing the irreplaceable role of this residue. Furthermore, paralogous validation in ZmHsf05 (R93A) confirms that this mechanism extends beyond individual Hsf members, providing a unified model for Hsf-DNA interaction within gene families.

The specificity of HSE recognition transcends core sequence fidelity. While canonical GAA repeats are essential, flanking sequences critically modulate binding efficiency [29]. Our EMSA analyses demonstrate that ZmHsf17 selectively binds HSE2 in the *ZmPAH1* promoter, despite the presence of the additional HSE motif (HSE1). Chimeric HSE constructs revealed that flanking sequences, particularly those upstream (HSE2L16) and downstream (HSE2R16), dictate recognition specificity, whereas the inter-GAA spacer (HSE2M2) plays a negligible role (Figure 5). These observations align with studies proposing that GC-rich flanking regions enhance DNA flexibility or stabilize minor groove interactions, thereby facilitating DBD engagement [9]. While our in vitro assays elucidate fundamental binding mechanisms, they cannot fully replicate the chromatin context in vivo. Prior work demonstrates that certain high-affinity HSEs in vitro remain unbound in planta, likely due to nucleosome positioning or epigenetic modifications [30]. Future studies employing ChIP-seq or live-cell imaging in transgenic maize will clarify how chromatin dynamics modulate ZmHsf17 targeting. Additionally, the functional divergence between typical (nGAAnnTTCn) and non-typical HSEs warrants exploration, particularly regarding their capacity to recruit Hsf under stress gradients [31].

The evolutionary conservation of the Hsf-DBD across divergent lineages, as evidenced by our phylogenetic analysis, highlights the indispensable role of DBD in maintaining functional integrity under selective pressure. The invariant “RQLN” motif within the α3 helix, conserved from thermophilic archaea to angiosperms, aligns with recent studies demonstrating its critical role in stabilizing DNA–protein interactions [25]. Notably, the preservation of key residues in α1 (K), β1 (W), and α2 (F, L, P) across 24 species suggests a universal structural scaffold for stress signal transduction, corroborating findings in mung bean HSFs where mutations in these regions impair thermotolerance [12]. This conservation contrasts with the lineage-specific divergence in AHA domains, which likely reflects adaptive evolution to niche-specific stressors. The phylogenetic segregation of plants, animals, and fungi into distinct clades underscores functional diversification in Hsf signaling. The conserved β4 sheet may play a role in coordinating abiotic stress responses unique to land plants. Beyond the conserved recognition helix, structural and functional conservation analyses identify additional critical residues for Hsf-DNA interaction; asparagine (N66 in HSF2/N74 in HSF1) and arginine (Arg109 in HSF2/Arg117 in HSF1) are evolutionarily conserved across Hsf orthologs [28]. These residues stabilize DNA binding through polar interactions with the phosphate backbone and base-specific hydrogen bonding, as evidenced by phylogenetic analyses and mutagenesis studies in yeast and mammalian systems [28]. Studies in tomato reveal that an arginine positioned within the β3-β4 loop region of the DBD plays auxiliary roles in stabilizing HSE engagement through structural stabilization rather than direct nucleotide contact [32]. The existing studies suggest that certain conserved residues mediate DNA-binding competency via conformational maintenance of the winged helix–turn–helix architecture.

By pinpointing Arg105 as a linchpin residue for HSE recognition and dissecting the interplay between cores and flanking sequences, this study advances our understanding of plant Hsf-DNA interaction mechanisms. The evolutionary conservation of arginine-mediated DNA binding across kingdoms underscores its fundamental role in stress adaptation. These insights not only refine molecular models of thermotolerance but also provide actionable targets for precision engineering of heat-resilient crops through residue-specific Hsf optimization.

## 4. Materials and Methods

### 4.1. Plant Materials and Gene Cloning

The coding sequences (CDS) of *ZmHsf17* and *ZmHsf05* were amplified from the maize (*Zea mays*) inbred line H21. A 2 kb promoter region of *ZmPAH1* was cloned from the *ZmHsf17* overexpressing line (receptor: Zheng58 inbred line) using primers designed based on our previous study [20].

### 4.2. Protein–DNA Docking via AlphaFold 3

Protein sequences (ZmHsf17 and ZmHsf05) and the *ZmPAH1* promoter sequence were formatted as FASTA files and uploaded to the DeepMind AlphaFold Server https://alphafoldserver.com/ (accessed on 10 December 2024) for molecular docking. The top-ranked model by confidence score was selected for structural analysis. Protein–DNA interaction interfaces, including hydrogen-bonding residues and nucleotide positions, were visualized using PyMOL 2.5. The structural model generated by AlphaFold 3 in CIF format was imported into PyMOL 2.5 for visualization. Hydrogen-bonding networks between proteins and DNA were analyzed using PyMOL’s built-in hydrogen bond detection algorithm, which identifies potential donor–acceptor pairs based on geometric criteria (bond length ≤ 3.5 Å, bond angle ≥ 120°).

### 4.3. Site-Directed Mutagenesis by Overlap Extension PCR

Critical residues in ZmHsf17 (R105, T109, K142) and ZmHsf05 (R93) were substituted with alanine to investigate their functional roles in DNA binding. Target codons for mutagenesis were mapped using the ExPASy 3.0 Translate tool https://web.expasy.org/translate/ (accessed on 15 December 2024) with standard genetic code parameters. Mutation-bearing primer pairs (F1/R1 and F2/R2) were designed using PrimerX 1.0 https://www.bioinformatics.org/primerx/ (accessed on 15 December 2024) with the following constraints: Tm = 62 ± 2 °C, GC content = 45–60%, and 15 bp overlapping regions flanking the mutation sites (Table S1). First-round PCR amplification generated upstream and downstream fragments with overlapping mutation sites. These fragments were mixed as templates for a second PCR using flanking primers (F1/R2). Amplified products were gel-purified and validated by Sanger sequencing (Sangon, Shanghai, China).

### 4.4. Recombinant Protein Expression and Purification

Wild-type and mutant CDS were cloned into the pET-30a vector (P3120, Solarbio, Beijing, China) with a C-terminal 6 × His tag via homologous recombination, and the specific steps for conducting the experiment should be referred to in the instructions provided with the reagent kit (C112, Vazyme, Nanjing, China). Chemically competent *E. coli* BL21 (DE3) cells (SP8171, Pyeast, Wuhan, China) were transformed via heat shock (42 °C, 45 s) and selected on LB agar plates supplemented with 50 μg/mL kanamycin at 37 °C for 16 h. For protein production, a single positive colony was inoculated into 5 mL LB-Kan medium and cultured overnight (37 °C, 220 rpm). The starter culture was diluted 1:100 into 500 mL LB-Kan medium and grown to the mid-log phase (OD600 = 0.6–0.8) at 37 °C with vigorous aeration. Protein expression was induced by adding 0.5 mmol/L isopropyl β-D-1-thiogalactopyranoside (IPTG), followed by incubation at 16 °C for 20 h with reduced agitation (180 rpm) to minimize inclusion body formation.

Cells were harvested by centrifugation (4 °C, 5000× *g*, 10 min) and resuspended in lysis buffer (20 mmol/L Tris-HCl pH 8.0, 500 mmol/L NaCl, 10 mmol/L imidazole, 6 mol/L guanidine hydrochloride). After sonication (Power 400 W, probe diameter 6 mm, 30% amplitude, 5 s on/10 s off, 30 min total), the lysate was clarified by centrifugation (12,000× *g*, 30 min) and filtered through 0.45 μm PVDF membranes. His-tagged proteins were purified using Ni-NTA affinity chromatography (C600033, Sangon) under denaturing conditions as described [20]. His-tagged proteins were purified under denaturing conditions with the following gradient: Wash 1: 5 mL metal chelating affinity chromatography (MCAC) buffer (20 mmol/L Tris-HCl, 500 mmol/L NaCl, 10 mmol/L β-Mercaptoethanol, 10% Glycerol); Elution 1: 5 mL MCAC buffer with 20 mmol/L imidazole; Elution 2: 5 mL MCAC buffer with 50 mmol/L imidazole; Elution 3: 5 mL MCAC buffer with 80 mmol/L imidazole; Elution 4: 5 mL MCAC buffer with 100 mmol/L imidazole; Elution 5: 5 mL MCAC buffer with 200 mmol/L imidazole. Protein concentration was determined by Bradford assay (Bio-Rad Laboratories, Shanghai, China) with BSA standards, and purity was confirmed by 12% SDS-PAGE with Coomassie Brilliant Blue R-250 staining.

### 4.5. Electrophoretic Mobility Shift Assay (EMSA)

Biotinylated double-stranded DNA probes mimicking the canonical HSE motif (5′-GAANNTTC-3′) were designed based on the *ZmPAH1* promoter region and its variants (mutant/core-flanking chimeras) were synthesized (GS008, Beyotime, Shanghai, China). Follow these steps for annealing in the PCR machine: 95 °C for 2 min, followed by ramp cooling to 25 °C at 0.1 °C/s. DNA–protein complexes were detected using a Chemiluminescent EMSA Kit (GS009, Beyotime). DNA–protein binding reactions (10 μL final volume) were assembled with: biotinylated an HSE probe, a mutation HSE probe, a purified recombinant protein, an EMSA/Gel-Shift buffer, a competitive probe, a competitive mutation HSE probe, and nuclease-free water. Reactions were incubated at 25 °C for 30 min before loading onto 6% non-denaturing polyacrylamide gels (29:1 acrylamide: bis ratio) pre-run in 0.5× TBE buffer (45 mmol/L Tris-borate, 1 mmol/L EDTA) at 100 V for 30 min. Electrophoresis was performed at 4 °C with a constant voltage (80 V) for 90 min. Nucleic acid transfer to positively charged nylon membranes (FFN11, Beyotime) was conducted via wet blotting (380 mA, 30 min). Biotin-labeled complexes were detected using the chemiluminescent EMSA Kit (GS009, Beyotime) following the manufacturer’s protocols. The band intensity was visualized using the Tanon 5200 Imaging System (Tanon, Shanghai, China). Probe sequences and mutagenesis designs are detailed in Table S1.

### 4.6. Phylogenetic Tree of Hsf-DBD Regions in 25 Species

The DBDs of ZmHsf05 and ZmHsf17 were subjected to comparative sequence analysis in the NCBI non-redundant protein database through BLASTp. Twenty-four phylogenetically representative species spanning key evolutionary nodes (11 plants, 8 metazoans, 5 fungi, 1 archaeon) were selected based on taxonomic significance and sequence similarity (>80% coverage). The multiple sequence alignment (MSA) of the retrieved DBD sequences was performed using MUSCLE v3.8.31 integrated in MEGA 7.0 with default parameters (gap opening penalty: −400, gap extension: 0). The alignment quality was validated by GUIDANCE2 scores (>0.93 across all positions). Phylogenetic reconstruction was then conducted using MEGA 7.0 via the maximum likelihood (ML) method with 1000 bootstrap replicates, applying the Jones–Taylor–Thornton (JTT) substitution model and gamma-distributed rate heterogeneity (α = 0.85). Sequence conservation patterns were visualized using WebLogo 3.0. The curated MSA file in FASTA format was uploaded to https://weblogo.berkeley.edu/ (accessed on 29 December 2024), generating sequence logos with Shannon entropy-based bit scores calculated for each position.

## Data Availability

All data are contained within the article and Supplementary Materials.

## Supplementary Materials

The following supporting information can be downloaded at: https://www.mdpi.com/article/10.3390/plants14131950/s1, Figure S1. HSE1 of *ZmPAH1* promoter cannot be bound by ZmHsf17 and ZmHsf05; Table S1. The sequences of all the primers and DNA probes.
