# Supplementary material for: The Structural Deciphering of the α3 Helix Within ZmHsfA2’S DNA-Binding Domain for the Recognition of Heat Shock Elements in Maize
Source: Plants (Basel). 2025 Jun 25;14(13):1950. doi: 10.3390/plants14131950 (PMC12251660; doi:10.3390/plants14131950)
Supplement: Supplementary file 1 [file plants-14-01950-s001.zip › Supplement files/Table S1.pdf]

Table S1 The sequences of all the primers and DNA probes.

Primers/probes	Forward sequence (5'–3')	Reverse sequence (5'–3')
ZmHsf17	ATGGACTCAACGCTGAAC	CTAATTGCTAGATGTTGG
ZmHsf17-R105A	GCTTCGTCGCGCAGCTCA	TGAGCTGCGCGACGAAGC
ZmHsf17-T109A	GCTCAACGCCTATGGTTTC	GAAACCATAGGCGTTGAGC
ZmHsf17-K142A	GCGTCGAGCACCTCCTGC	GCAGGAGGTGCTCGACGC
ZmHsf17-pET30a	GCTGATATCGGATCCGAATTCATGGACTCAACGCTGAAC	GCGGCCGCAAGCTTGTCGACCTAATTGCTAGATGTTG
ZmHsf05	ATGAGCCACGGGAACGGG	CTAATGTCTGGGTCCATGTT
ZmHsf05-R93A	GCTTTGTTGCGCAGCTCA	GAGCTGCGCAACAAAG
ZmHsf05-pET30a	TATCGGATCCGAATTCATGAGCCACGGG	GCGGCCGCAAGCTTGTCGACATGTCTGGGTCC
ZmPAH1-HSE1	TCTTCGAATTGCATAGGAAACTTCGTTCGAACGCATTCTG	CAGAATGCGTTCGAACGAAGTTTCCTATGCAATTCGAAGA
ZmPAH1-mutantHSE1	TCTTCGAATTGCATAGTAAACTTAGTTCGAACGCATTCTG	CAGAATGCGTTCGAACTAAGTTTACTATGCAATTCGAAGA
ZmPAH1-HSE2	CAGCGTCACATGACGGGAATTTTCACGTCTTTTCAGCCCT	AGGGCTGAAAAGACGTGAAAATTCCCGTCATGTGACGCTG
ZmPAH1-mutantHSE2	CAGCGTCACATGACGGAAATTTTAACGTCTTTTCAGCCCT	AGGGCTGAAAAGACGTTAAAATTTCCGTCATGTGACGCTG
ZmPAH1-HSE1-HSE2L16	CAGCGTCACATGACGGGAATTTTCGTTCGAACGCATTCTG	CAGAATGCGTTCGAACGAAAATTCCCGTCATGTGACGCTG
ZmPAH1-HSE1-HSE2M2	TCTTCGAATTGCATAGGAATTTTCGTTCGAACGCATTCTG	CAGAATGCGTTCGAACGAAAATTCCTATGCAATTCGAAGA
ZmPAH1-HSE1-HSE2R16	TCTTCGAATTGCATAGGAAACTTCACGTCTTTTCAGCCCT	AGGGCTGAAAAGACGTGAAGTTTCCTATGCAATTCGAAGA
