# Supplementary figures and images for: The Structural Deciphering of the α3 Helix Within ZmHsfA2’S DNA-Binding Domain for the Recognition of Heat Shock Elements in Maize
Source: Plants (Basel). 2025 Jun 25;14(13):1950. doi: 10.3390/plants14131950 (PMC12251660; doi:10.3390/plants14131950)

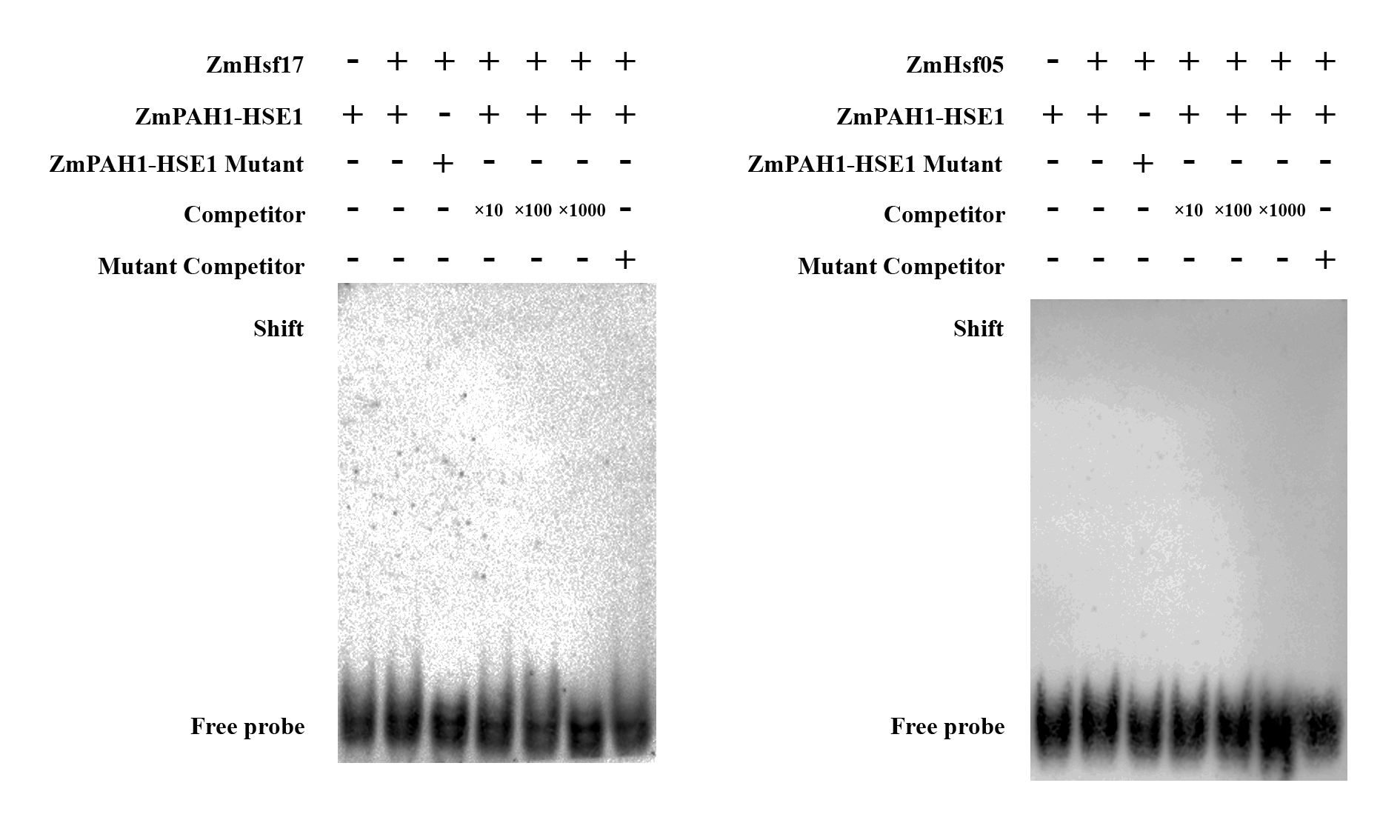

Supplement: Supplementary file 1 [file plants-14-01950-s001.zip › Supplement files/Figure S1.tif]
